# Crystal structure of the charge-transfer complex 2-(1,2,3,4-tetra­hydro­naph­thal­en-1-yl­idene)hydrazinecarbo­thio­amide–pyrazine-2,3,5,6-tetra­carbo­nitrile (2/1)

**DOI:** 10.1107/S1600536814019795

**Published:** 2014-09-06

**Authors:** Johannes Beck, Jörg Daniels, Petra Krieger-Beck, Gertrud Dittmann, Adriano Bof de Oliveira

**Affiliations:** a Institut für Anorganische Chemie, Universität Bonn, Gerhard-Domagk-Strasse 1, D-53121 Bonn, Germany; b Departamento de Química, Universidade Federal de Sergipe, Av. Marechal Rondon s/n, Campus, 49100-000 São Cristóvão-SE, Brazil

**Keywords:** charge-transfer composite compound, tetra­cyano­pyrazine, thio­semicarbazone, crystal structure

## Abstract

The reaction of 2-(1,2,3,4-tetra­hydro­napthalen-1-yl­idene)hydrazinecarbo­thio­amide (TTSC) with pyrazine-2,3,5,6-tetra­carbo­nitrile (tetra­cyano­pyrazine, TCNP) yields the title 2:1 charge-transfer adduct, 2C_11_H_12_N_3_S·C_6_N_8_. The complete TCNP mol­ecule is generated by a crystallographic inversion centre and the non-aromatic ring in the TTSC mol­ecule adopts an envelope conformation with a methyl­ene C atom as the flap. In the crystal, the thio­semicarbazone mol­ecules are connected through inversion-related pairs of N—H⋯S inter­actions, building a polymeric chain along the *b*-axis direction. The TCNP mol­ecules are embedded in the structure, forming TTSC–TCNP–TTSC stacks with the aromatic rings of TTSC and the mol­ecular plane of TCNP in a parallel arrangement [centroid–centroid distance = 3.5558 (14) Å]. Charge-transfer (CT) *via* π-stacking is indicated by a CT band around 550 cm^−1^ in the single-crystal absorption spectrum.

## Related literature   

For one of the first reports of the synthesis of thio­semicarbazone derivatives, see: Freund & Schander (1902 ▶). For the crystal structure of tetra­lone–thio­semicarbazone, see: de Oliveira *et al.* (2012 ▶7). For charge-transfer compounds involving TCNP, see: Rosokha *et al.* (2004 ▶). Tetra­cyano­pyrazine was obtained by condensation of di­imino­succino­nitrile with di­amino­maleo­nitrile according to a literature procedure (Begland *et al.*, 1974 ▶) For bond lengths in neat TCNP, see: Rosokha *et al.* (2009 ▶) and for the electronic situation in the TCNP mol­ecule, see: Novoa *et al.* (2009 ▶).
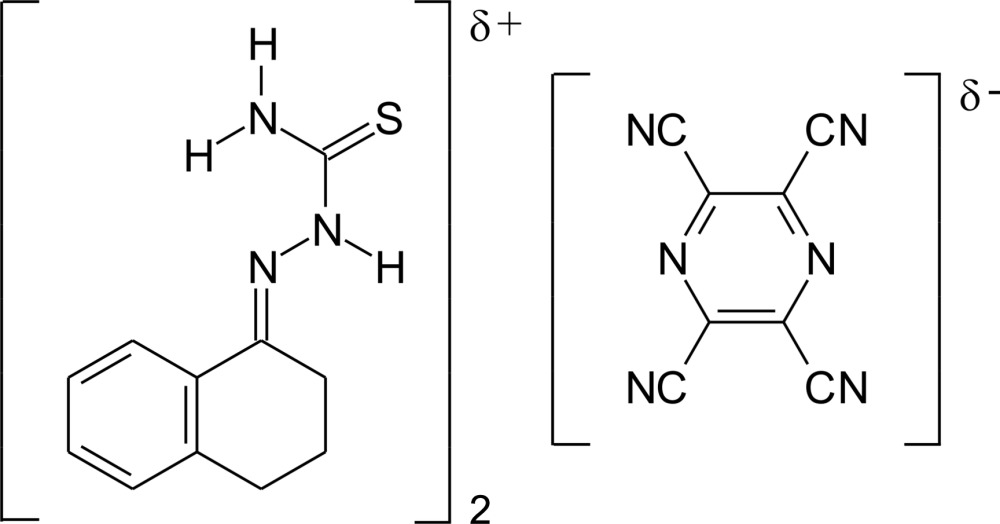


## Experimental   

### Crystal data  

2C_11_H_13_N_3_S·C_8_N_6_
*M* *_r_* = 618.74
Triclinic, 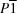

*a* = 6.1363 (4) Å
*b* = 8.2574 (3) Å
*c* = 15.3303 (9) Å
α = 86.659 (3)°
β = 78.751 (2)°
γ = 73.893 (3)°
*V* = 731.95 (7) Å^3^
*Z* = 1
Mo *K*α radiation
μ = 0.23 mm^−1^
*T* = 293 K
0.06 × 0.04 × 0.02 mm

### Data collection  

Nonius KappaCCD diffractometer
Absorption correction: analytical (Alcock, 1970 ▶) *T*_min_ = 0.987, *T*_max_ = 0.995
10255 measured reflections
2601 independent reflections
1775 reflections with *I* > 2σ(*I*)
*R*_int_ = 0.073

### Refinement  

*R*[*F* ^2^ > 2σ(*F* ^2^)] = 0.045
*wR*(*F* ^2^) = 0.096
*S* = 1.08
2601 reflections
251 parameters
All H-atom parameters refined
Δρ_max_ = 0.26 e Å^−3^
Δρ_min_ = −0.31 e Å^−3^

### 

Data collection: *COLLECT* (Nonius, 1998 ▶); cell refinement: *SCALEPACK* (Otwinowski & Minor, 1997 ▶); data reduction: *DENZO* (Otwinowski & Minor, 1997 ▶) and *SCALEPACK*; program(s) used to solve structure: *SHELXS97* (Sheldrick, 2008 ▶); program(s) used to refine structure: *SHELXL97* (Sheldrick, 2008 ▶); molecular graphics: *DIAMOND* (Brandenburg, 2006 ▶); software used to prepare material for publication: *publCIF* (Westrip, 2010 ▶).

## Supplementary Material

Crystal structure: contains datablock(s) I, publication_text. DOI: 10.1107/S1600536814019795/hb7278sup1.cif

Structure factors: contains datablock(s) I. DOI: 10.1107/S1600536814019795/hb7278Isup2.hkl

**Fig. 1.**
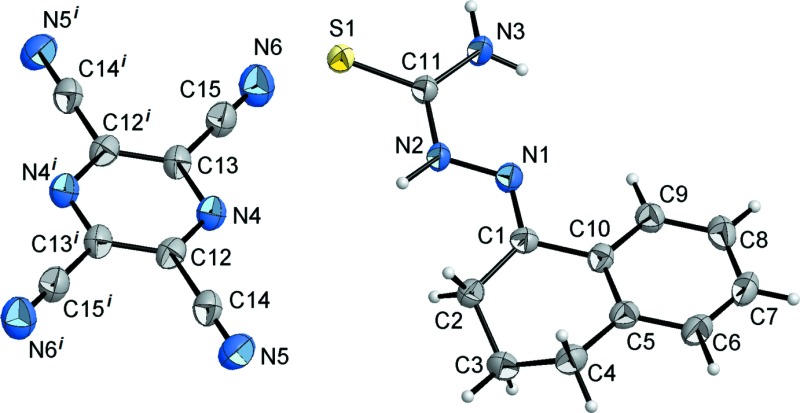
: The two molecular constituents of the title compound with displacement ellipsoids drawn at the 70% probability level. Symmetry code: (i) 1- x, 1 - y ,-z.

**Fig. 2.**
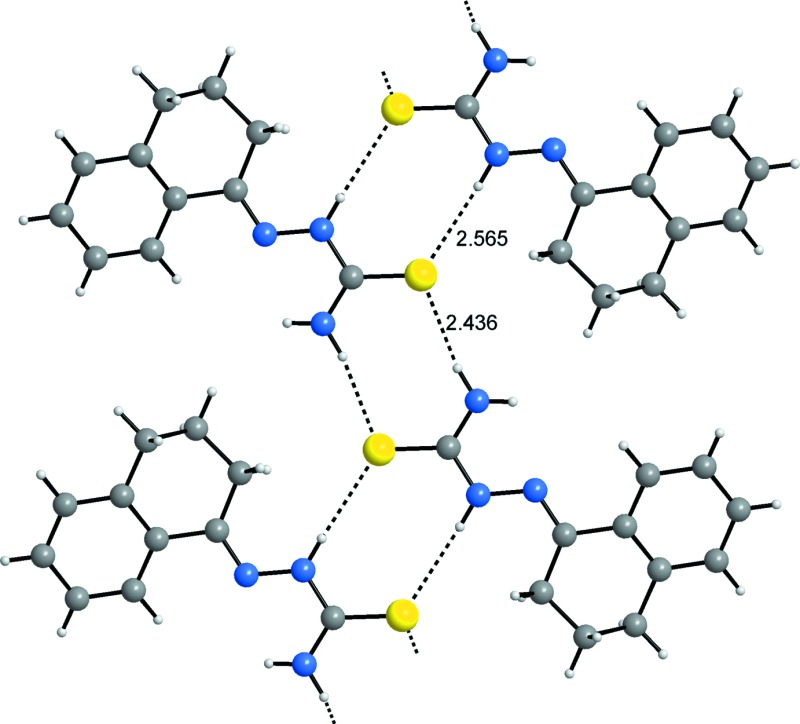
: Molecules of TTSC connected via N—H···S hydrogen bridges to an infinite ribbon. Bond lengths are given in Å.

**Fig. 3.**
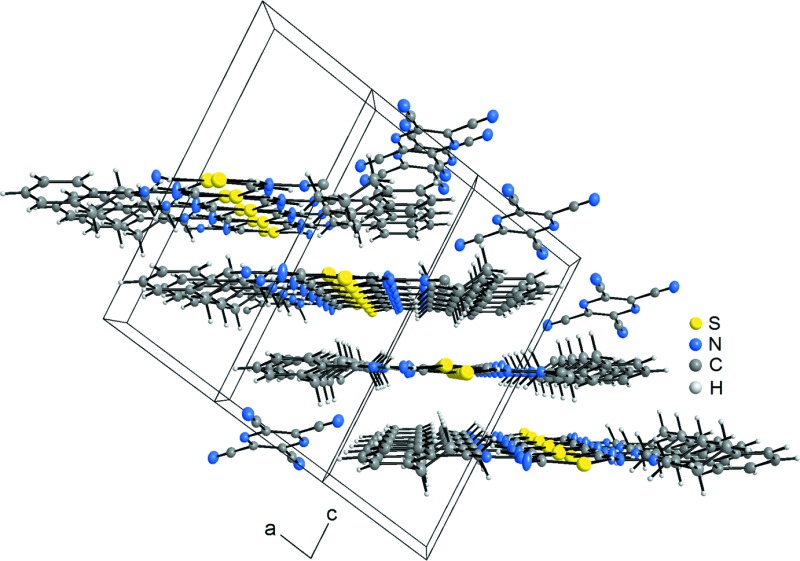
: The arrangement of the molecules in the structure of the title compound in a perspective view along the b-axis.

Supporting information file. DOI: 10.1107/S1600536814019795/hb7278Isup3.cml

x y z . DOI: 10.1107/S1600536814019795/hb7278fig1.tif
The two mol­ecular constituents of the title compound with displacement ellipsoids drawn at the 70% probability level. Symmetry code: (i) 1 − *x*, 1 − *y*, -*z*.

via . DOI: 10.1107/S1600536814019795/hb7278fig2.tif
Mol­ecules of TTSC connected *via* N—H⋯S hydrogen bridges to an infinite ribbon. Bond lengths are given in Å.

b . DOI: 10.1107/S1600536814019795/hb7278fig3.tif
The arrangement of the mol­ecules in the structure of the title compound in a perspective view along the *b*-axis.

**Fig. 4.**
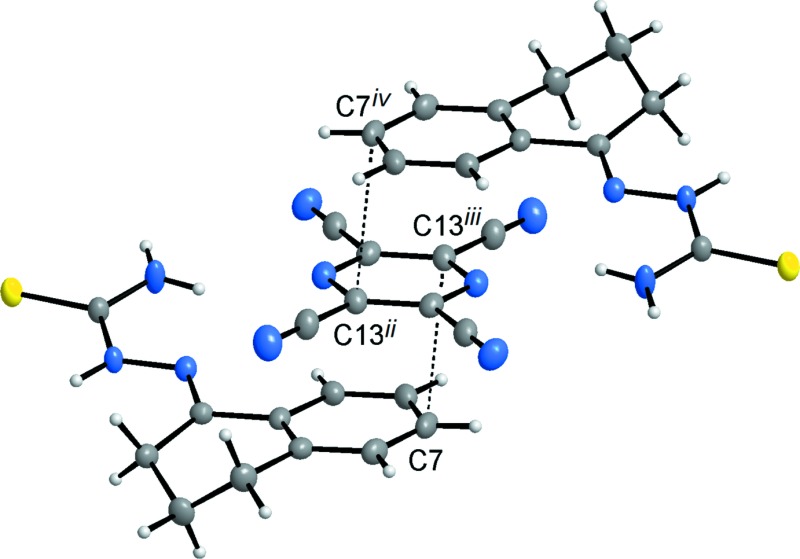
: Detail of the crystal structure of the title compound (TTSC)2TCNP). The TCNP molecules are embedded between two phenyl rings of adjacent TTSC molecules. The shortest distance amounts to C7···C13iii = 3.233 Å. Symmetry codes: (ii)-x,1 - y,1 - z, (iii)1 - x,y,1 + z, (iv)-1 - x,1 - y,2 - z.

2 iii x y z x y z x y z . DOI: 10.1107/S1600536814019795/hb7278fig4.tif
Detail of the crystal structure of the title compound (TTSC)_2_TCNP). The TCNP mol­ecules are embedded between two phenyl rings of adjacent TTSC mol­ecules. The shortest distance amounts to C7⋯C13^iii^ = 3.233 Å. Symmetry codes: (ii)-*x*,1 − *y*,1 − *z*, (iii)1 − *x*,*y*,1 + *z*, (iv)-1 − *x*,1 − *y*,2 − *z*.

**Fig. 5.**
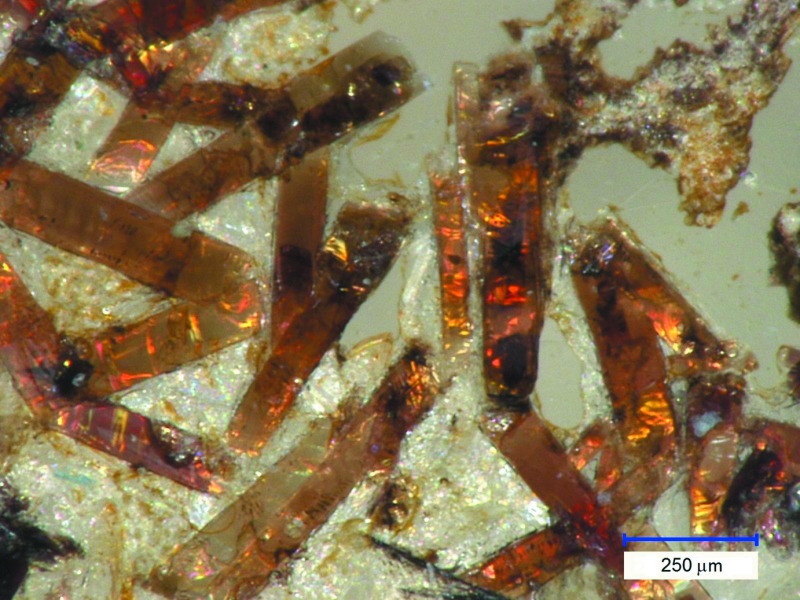
: Photo of crystals of the title compound. The crystals are embedded in unreacted TCNP, which was used in excess.

. DOI: 10.1107/S1600536814019795/hb7278fig5.tif
Photo of crystals of the title compound. The crystals are embedded in unreacted TCNP, which was used in excess.

**Fig. 6.**
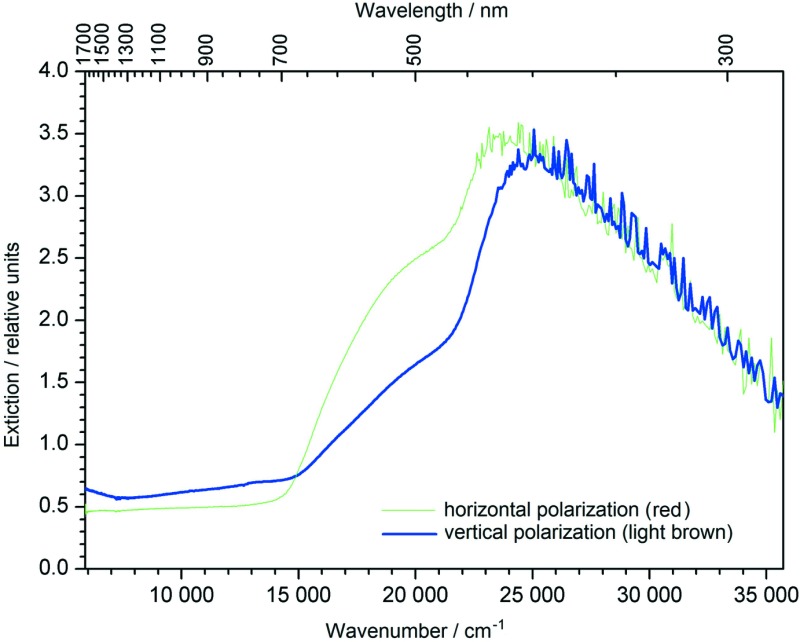
: Crystal UV-vis absorption spectrum of the title compound recorded with light in horizontal and vertical polarization direction.

. DOI: 10.1107/S1600536814019795/hb7278fig6.tif
Crystal UV-vis absorption spectrum of the title compound recorded with light in horizontal and vertical polarization direction.

CCDC reference: 1022549

Additional supporting information:  crystallographic information; 3D view; checkCIF report

## Figures and Tables

**Table 1:** Hydrogen-bond geometry (Å, °)

*D*—H⋯*A*	*D*—H	H⋯*A*	*D*⋯*A*	*D*—H⋯*A*
N2—H*N*2⋯S1^i^	0.89 (3)	2.57 (3)	3.450 (2)	173 (2)
N3—H*N*3*A*⋯S1^ii^	0.92 (3)	2.44 (3)	3.348 (2)	170 (3)

Symmetry codes: (i) 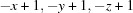; (ii) 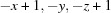.

## supplementary crystallographic information

### S2. Structural commentary

The title compound represents a charge-transfer adduct composite of tetra­hydro­naphtalene-thio­semicarbazone (TTSC) and the strong electron acceptor pyrazine­tetra­carbo­nitrile (tetra­cyano­pyrazine, TCNP) in stoichiometry 2:1 (Fig. 1). TTSC has the maximum deviation from the mean plane of the non-H atoms of 0.448 (2) Å for C3, which corresponds to an envelope conformation for the non-aromatic ring. The structure of the thio­semicarbazone derivative is quite similar to the structure reported in the literature (Oliveira *et al.*, 2012). The molecule shows an *E* conformation for the atoms about the N1–N2 bond. The torsion angle at the atoms N1, N2, C11 and S1 amounts to 176.4 (2)°, building a slightly distorted planar environment. The molecules are connected through inversion centres via pairs of N1–H···S inter­actions (Fig. 2 and Table 1) forming a one-dimensional hydrogen-bonded polymer running along the *b*-axis (Fig. 2).

The TCNP molecule is essentially planar with the maximum deviation of 0.006 Å from the least squares plane through all atoms. Bond lengths differ less than 0.01 Å to neat TCNP (Rosokha *et al.* 2009). These non-significant differences show that the amount of charge-transfer is comparably small and the electronic situation of the TCNP molecule is mainly unaltered (Novoa *et al.*, 2009).

The molecular planes of TTSC and TCNP molecules are parallel and arranged perpendicular to the [101] direction (Fig. 3). The TCNP molecules from stacks with each two neighboring TTSC molecules. The aromatic rings of two TTSC molecules and the TCNP planes are in an almost parallel arrangement. The shortest distances between the six-membered rings of 3.233 Å are observed between C7 of TTSC and C13 of TCNP (Fig. 4). As typical for weak to medium strong charge-transfer complexes between π systems, the normals through the midpoints of the aromatic rings are not coincident, instead the stack is slipped by about 15°.

The presence of a substantial charge-transfer in the title compound is indicated by the red colour, since the starting materials are light-yellow (TTSC) and colourless (TCNP) (Fig. 5). Moreover, the crystals are dichroitic and show a colour change in polarized light from red to light-brown.

In the single crystal absorption spectrum, the charge-transfer band is present in the range 500 -650 nm (Fig. 6). Depending on the incident angle of the plane of polarization a distinct change of the charge-transfer band both in intensity and energy is present, explaining the different colours.

### S5. Synthesis and crystallization

The synthesis of 2-(1,2,3,4-tetra­hydro­naphthalen-1-yl­idene)hydrazinecarbo­thio­amide was adapted from a procedure reported over 100 years' ago (Freund & Schander, 1902). Tetra­cyano­pyrazine was obtained by condensation of di­imino­succino­nitrile with di­amino­maleo­nitrile according to literature (Begland *et al.*, 1974). The title compound (TTSC)_2_(TCNP) is formed only if an excess of TCNP is present. Solutions of two molar equivalents of TNCP and of one molar equivalent of TTSC in aceto­nitrile are prepared. On mixing the solutions, no significant colour change is observed. Slow evaporation of the solvent affords crystals of (TTSC)_2_(TCNP) as thin light-red plates, embedded in a matrix of excess TCNP. Crystals of the title compound had to be separated mechanically.

### S6. Refinement

Crystal data, data collection and structure refinement details are summarized in Table 1. All hydrogen atoms were localized in a difference density Fourier map. Their positions and isotropic displacement parameters were refined.

### Crystal data

**Table:**

2C_11_H_13_N_3_S·C_8_N_6_	*Z* = 1
*M**_r_* = 618.74	*F*(000) = 322
Triclinic, *P*1	*D*_x_ = 1.404 Mg m^−^^3^
Hall symbol: -P 1	Mo *K*α radiation, λ = 0.71073 Å
*a* = 6.1363 (4) Å	Cell parameters from 15334 reflections
*b* = 8.2574 (3) Å	θ = 2.9–27.5°
*c* = 15.3303 (9) Å	µ = 0.23 mm^−^^1^
α = 86.659 (3)°	*T* = 293 K
β = 78.751 (2)°	Plate, red
γ = 73.893 (3)°	0.06 × 0.04 × 0.02 mm
*V* = 731.95 (7) Å^3^	

### Data collection

**Table:**

Nonius KappaCCD diffractometer	2601 independent reflections
Radiation source: fine-focus sealed tube, Nonius KappaCCD	1775 reflections with *I* > 2σ(*I*)
Graphite monochromator	*R*_int_ = 0.073
Detector resolution: 9 pixels mm^-1^	θ_max_ = 25.1°, θ_min_ = 3.5°
CCD rotation images, thick slices scans	*h* = −7→7
Absorption correction: analytical (Alcock, 1970)	*k* = −9→9
*T*_min_ = 0.987, *T*_max_ = 0.995	*l* = −18→18
10255 measured reflections	

### Refinement

**Table:**

Refinement on *F*^2^	Primary atom site location: structure-invariant direct methods
Least-squares matrix: full	Secondary atom site location: difference Fourier map
*R*[*F*^2^ > 2σ(*F*^2^)] = 0.045	Hydrogen site location: inferred from neighbouring sites
*wR*(*F*^2^) = 0.096	All H-atom parameters refined
*S* = 1.08	*w* = 1/[σ^2^(*F*_o_^2^) + (0.0321*P*)^2^ + 0.1427*P*] where *P* = (*F*_o_^2^ + 2*F*_c_^2^)/3
2601 reflections	(Δ/σ)_max_ < 0.001
251 parameters	Δρ_max_ = 0.26 e Å^−^^3^
0 restraints	Δρ_min_ = −0.31 e Å^−^^3^

### Special details

**Table:**

Geometry. All e.s.d.'s (except the e.s.d. in the dihedral angle between two l.s. planes) are estimated using the full covariance matrix. The cell e.s.d.'s are taken into account individually in the estimation of e.s.d.'s in distances, angles and torsion angles; correlations between e.s.d.'s in cell parameters are only used when they are defined by crystal symmetry. An approximate (isotropic) treatment of cell e.s.d.'s is used for estimating e.s.d.'s involving l.s. planes.
Refinement. Refinement of *F*^2^ against ALL reflections. The weighted *R*-factor *wR* and goodness of fit *S* are based on *F*^2^, conventional *R*-factors *R* are based on *F*, with *F* set to zero for negative *F*^2^. The threshold expression of *F*^2^ > σ(*F*^2^) is used only for calculating *R*-factors(gt) *etc*. and is not relevant to the choice of reflections for refinement. *R*-factors based on *F*^2^ are statistically about twice as large as those based on *F*, and *R*- factors based on ALL data will be even larger.

### Fractional atomic coordinates and isotropic or equivalent isotropic displacement parameters (Å^2^)

**Table:**

	*x*	*y*	*z*	*U*_iso_*/*U*_eq_	
S1	0.57106 (12)	0.24556 (8)	0.49235 (5)	0.0255 (2)	
N1	−0.0374 (4)	0.4493 (2)	0.63392 (13)	0.0209 (5)	
N2	0.1759 (4)	0.4342 (3)	0.58009 (13)	0.0202 (5)	
N3	0.2050 (4)	0.1530 (3)	0.57790 (17)	0.0311 (6)	
C1	−0.1576 (4)	0.5967 (3)	0.66381 (15)	0.0187 (6)	
C2	−0.0813 (5)	0.7547 (3)	0.64597 (19)	0.0218 (6)	
C3	−0.2709 (5)	0.9130 (3)	0.67964 (19)	0.0267 (6)	
C4	−0.3900 (5)	0.8872 (3)	0.77390 (18)	0.0271 (6)	
C5	−0.4936 (4)	0.7411 (3)	0.77777 (16)	0.0213 (6)	
C10	−0.3814 (4)	0.6016 (3)	0.72246 (15)	0.0191 (6)	
C9	−0.4809 (4)	0.4679 (3)	0.72410 (17)	0.0226 (6)	
C8	−0.6878 (5)	0.4710 (3)	0.78065 (17)	0.0260 (6)	
C7	−0.7975 (5)	0.6088 (3)	0.83626 (19)	0.0274 (6)	
C6	−0.7026 (5)	0.7431 (3)	0.83434 (17)	0.0265 (6)	
C11	0.3029 (4)	0.2765 (3)	0.55299 (16)	0.0203 (6)	
H2A	−0.030 (4)	0.763 (3)	0.5850 (16)	0.012 (6)*	
H2B	0.059 (5)	0.742 (3)	0.6765 (17)	0.030 (7)*	
H3A	−0.383 (5)	0.937 (3)	0.6388 (19)	0.039 (8)*	
H3B	−0.205 (4)	1.011 (3)	0.6756 (16)	0.028 (7)*	
H4A	−0.509 (5)	0.991 (3)	0.7973 (17)	0.034 (7)*	
H4B	−0.275 (5)	0.862 (3)	0.8164 (17)	0.032 (7)*	
H6	−0.404 (4)	0.373 (3)	0.6829 (17)	0.032 (7)*	
H7	−0.754 (5)	0.383 (3)	0.7821 (17)	0.030 (7)*	
H8	−0.933 (5)	0.612 (3)	0.8765 (19)	0.038 (8)*	
H9	−0.779 (5)	0.839 (3)	0.8738 (18)	0.035 (8)*	
HN2	0.242 (5)	0.518 (3)	0.5664 (18)	0.037 (8)*	
HN3A	0.283 (5)	0.047 (4)	0.5558 (19)	0.044 (9)*	
HN3B	0.065 (6)	0.176 (4)	0.607 (2)	0.053 (10)*	
N4	0.2849 (4)	0.4881 (3)	0.04821 (14)	0.0251 (5)	
N5	0.1068 (4)	0.8479 (3)	0.17016 (16)	0.0355 (6)	
N6	0.2570 (4)	0.1174 (3)	−0.02636 (15)	0.0375 (6)	
C12	0.3663 (4)	0.6193 (3)	0.05758 (16)	0.0228 (6)	
C13	0.4196 (4)	0.3699 (3)	−0.00991 (16)	0.0231 (6)	
C14	0.2226 (5)	0.7472 (3)	0.12048 (18)	0.0263 (6)	
C15	0.3294 (5)	0.2286 (3)	−0.01969 (18)	0.0282 (6)	

### Atomic displacement parameters (Å^2^)

**Table:**

	*U*^11^	*U*^22^	*U*^33^	*U*^12^	*U*^13^	*U*^23^
S1	0.0220 (4)	0.0187 (3)	0.0324 (4)	−0.0062 (3)	0.0051 (3)	−0.0043 (3)
N1	0.0186 (12)	0.0223 (11)	0.0218 (12)	−0.0064 (9)	−0.0019 (10)	−0.0011 (9)
N2	0.0174 (12)	0.0189 (12)	0.0232 (12)	−0.0073 (10)	0.0027 (10)	−0.0028 (9)
N3	0.0210 (15)	0.0194 (13)	0.0476 (16)	−0.0074 (11)	0.0105 (12)	−0.0064 (11)
C1	0.0198 (14)	0.0176 (13)	0.0185 (14)	−0.0044 (10)	−0.0040 (11)	−0.0006 (10)
C2	0.0202 (16)	0.0215 (14)	0.0207 (16)	−0.0030 (11)	0.0001 (13)	−0.0009 (11)
C3	0.0286 (17)	0.0185 (14)	0.0320 (17)	−0.0075 (12)	−0.0009 (14)	−0.0023 (11)
C4	0.0271 (17)	0.0229 (15)	0.0279 (16)	−0.0044 (12)	0.0007 (14)	−0.0047 (12)
C5	0.0207 (15)	0.0216 (13)	0.0198 (14)	−0.0029 (11)	−0.0044 (12)	0.0011 (10)
C10	0.0169 (14)	0.0227 (13)	0.0172 (14)	−0.0046 (11)	−0.0032 (11)	0.0004 (10)
C9	0.0221 (16)	0.0224 (14)	0.0244 (15)	−0.0076 (12)	−0.0049 (12)	0.0012 (11)
C8	0.0219 (16)	0.0306 (16)	0.0276 (16)	−0.0110 (13)	−0.0062 (13)	0.0067 (12)
C7	0.0171 (16)	0.0365 (16)	0.0242 (16)	−0.0033 (12)	0.0001 (13)	0.0035 (12)
C6	0.0223 (16)	0.0290 (15)	0.0233 (16)	−0.0011 (12)	−0.0007 (13)	−0.0016 (12)
C11	0.0186 (15)	0.0193 (13)	0.0224 (14)	−0.0064 (11)	0.0002 (11)	−0.0026 (10)
N4	0.0221 (13)	0.0290 (12)	0.0256 (13)	−0.0091 (10)	−0.0043 (10)	−0.0011 (10)
N5	0.0277 (15)	0.0353 (14)	0.0398 (16)	−0.0039 (11)	−0.0027 (12)	−0.0060 (12)
N6	0.0369 (16)	0.0411 (15)	0.0373 (15)	−0.0173 (13)	−0.0027 (12)	−0.0048 (11)
C12	0.0198 (15)	0.0278 (14)	0.0206 (14)	−0.0047 (11)	−0.0051 (12)	−0.0011 (11)
C13	0.0214 (15)	0.0282 (14)	0.0205 (14)	−0.0086 (12)	−0.0030 (12)	0.0000 (11)
C14	0.0217 (16)	0.0319 (15)	0.0267 (16)	−0.0098 (13)	−0.0042 (13)	0.0004 (12)
C15	0.0238 (16)	0.0319 (16)	0.0289 (16)	−0.0097 (13)	−0.0009 (13)	−0.0039 (12)

### Geometric parameters (Å, º)

**Table:**

S1—C11	1.684 (2)	C5—C6	1.398 (4)
N1—C1	1.292 (3)	C5—C10	1.402 (3)
N1—N2	1.382 (3)	C10—C9	1.399 (3)
N2—C11	1.360 (3)	C9—C8	1.385 (4)
N2—HN2	0.89 (3)	C9—H6	0.98 (3)
N3—C11	1.324 (3)	C8—C7	1.392 (4)
N3—HN3A	0.92 (3)	C8—H7	0.92 (3)
N3—HN3B	0.86 (3)	C7—C6	1.385 (4)
C1—C10	1.479 (3)	C7—H8	0.93 (3)
C1—C2	1.498 (3)	C6—H9	0.98 (3)
C2—C3	1.526 (3)	N4—C12	1.338 (3)
C2—H2A	0.93 (2)	N4—C13	1.340 (3)
C2—H2B	1.04 (3)	N5—C14	1.146 (3)
C3—C4	1.519 (4)	N6—C15	1.143 (3)
C3—H3A	0.99 (3)	C12—C13^i^	1.397 (4)
C3—H3B	0.99 (3)	C12—C14	1.447 (4)
C4—C5	1.505 (3)	C13—C12^i^	1.396 (4)
C4—H4A	0.99 (3)	C13—C15	1.450 (4)
C4—H4B	1.02 (3)		
			
C1—N1—N2	118.6 (2)	C6—C5—C4	121.2 (2)
C11—N2—N1	117.3 (2)	C10—C5—C4	119.6 (2)
C11—N2—HN2	117.5 (18)	C9—C10—C5	119.7 (2)
N1—N2—HN2	124.9 (18)	C9—C10—C1	120.8 (2)
C11—N3—HN3A	117.4 (19)	C5—C10—C1	119.6 (2)
C11—N3—HN3B	120 (2)	C8—C9—C10	120.6 (2)
HN3A—N3—HN3B	122 (3)	C8—C9—H6	120.5 (16)
N1—C1—C10	115.3 (2)	C10—C9—H6	118.9 (16)
N1—C1—C2	124.8 (2)	C9—C8—C7	119.6 (3)
C10—C1—C2	119.9 (2)	C9—C8—H7	120.9 (17)
C1—C2—C3	113.1 (2)	C7—C8—H7	119.5 (17)
C1—C2—H2A	108.4 (13)	C6—C7—C8	120.4 (3)
C3—C2—H2A	110.3 (14)	C6—C7—H8	118.5 (16)
C1—C2—H2B	107.8 (13)	C8—C7—H8	121.1 (17)
C3—C2—H2B	109.8 (13)	C7—C6—C5	120.6 (2)
H2A—C2—H2B	107 (2)	C7—C6—H9	120.3 (16)
C4—C3—C2	110.8 (2)	C5—C6—H9	119.0 (16)
C4—C3—H3A	110.5 (16)	N3—C11—N2	116.4 (2)
C2—C3—H3A	107.6 (15)	N3—C11—S1	123.34 (19)
C4—C3—H3B	111.5 (14)	N2—C11—S1	120.27 (18)
C2—C3—H3B	109.8 (15)	C12—N4—C13	116.1 (2)
H3A—C3—H3B	106 (2)	N4—C12—C13^i^	121.5 (2)
C5—C4—C3	110.5 (2)	N4—C12—C14	116.4 (2)
C5—C4—H4A	110.8 (15)	C13^i^—C12—C14	122.1 (2)
C3—C4—H4A	111.5 (15)	N4—C13—C12^i^	122.5 (2)
C5—C4—H4B	107.9 (14)	N4—C13—C15	115.6 (2)
C3—C4—H4B	110.8 (15)	C12^i^—C13—C15	121.9 (2)
H4A—C4—H4B	105 (2)	N5—C14—C12	179.3 (3)
C6—C5—C10	119.1 (2)	N6—C15—C13	179.2 (3)
			
C1—N1—N2—C11	177.1 (2)	N1—C1—C10—C5	162.0 (2)
N2—N1—C1—C10	−178.73 (19)	C2—C1—C10—C5	−15.5 (3)
N2—N1—C1—C2	−1.3 (3)	C5—C10—C9—C8	−0.8 (4)
N1—C1—C2—C3	172.5 (2)	C1—C10—C9—C8	178.4 (2)
C10—C1—C2—C3	−10.1 (3)	C10—C9—C8—C7	0.1 (4)
C1—C2—C3—C4	46.8 (3)	C9—C8—C7—C6	0.9 (4)
C2—C3—C4—C5	−58.8 (3)	C8—C7—C6—C5	−1.1 (4)
C3—C4—C5—C6	−144.3 (2)	C10—C5—C6—C7	0.3 (4)
C3—C4—C5—C10	34.5 (3)	C4—C5—C6—C7	179.1 (2)
C6—C5—C10—C9	0.6 (4)	N1—N2—C11—N3	2.8 (3)
C4—C5—C10—C9	−178.1 (2)	N1—N2—C11—S1	−176.37 (16)
C6—C5—C10—C1	−178.7 (2)	C13—N4—C12—C13^i^	−0.6 (4)
C4—C5—C10—C1	2.6 (3)	C13—N4—C12—C14	−179.7 (2)
N1—C1—C10—C9	−17.2 (3)	C12—N4—C13—C12^i^	0.6 (4)
C2—C1—C10—C9	165.2 (2)	C12—N4—C13—C15	−179.9 (2)

Symmetry code: (i) −*x*+1, −*y*+1, −*z*.

### Hydrogen-bond geometry (Å, º)

**Table:**

*D*—H···*A*	*D*—H	H···*A*	*D*···*A*	*D*—H···*A*
N2—H*N*2···S1^ii^	0.89 (3)	2.57 (3)	3.450 (2)	173 (2)
N3—H*N*3*A*···S1^iii^	0.92 (3)	2.44 (3)	3.348 (2)	170 (3)

Symmetry codes: (ii) −*x*+1, −*y*+1, −*z*+1; (iii) −*x*+1, −*y*, −*z*+1.
