# Supplementary figures and images for: Crystal structure of the charge-transfer complex 2-(1,2,3,4-tetra­hydro­naph­thal­en-1-yl­idene)hydrazinecarbo­thio­amide–pyrazine-2,3,5,6-tetra­carbo­nitrile (2/1)
Source: Acta Crystallogr Sect E Struct Rep Online. 2014 Sep 6;70(Pt 10):o1090–1. doi: 10.1107/S1600536814019795 (PMC4257178; doi:10.1107/S1600536814019795)

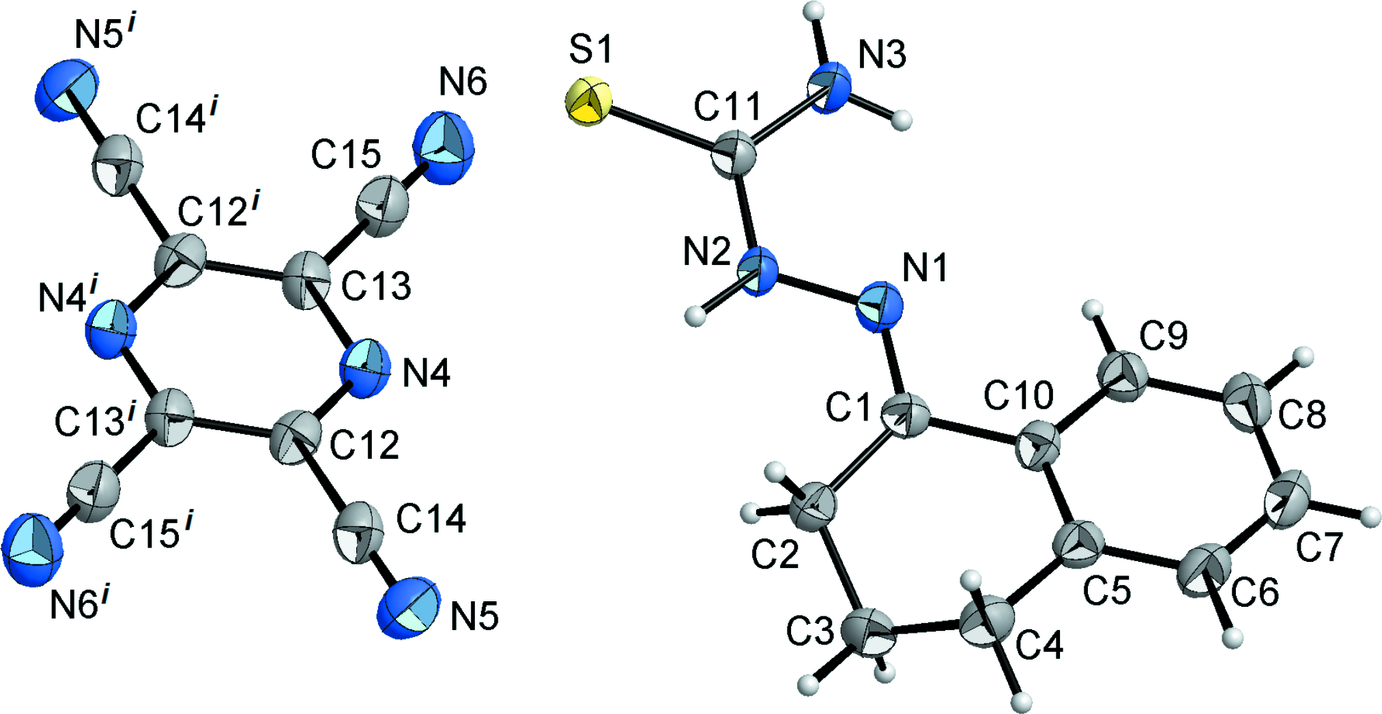

Supplement: Supplementary file 4 [file e-70-o1090-fig1.tif]

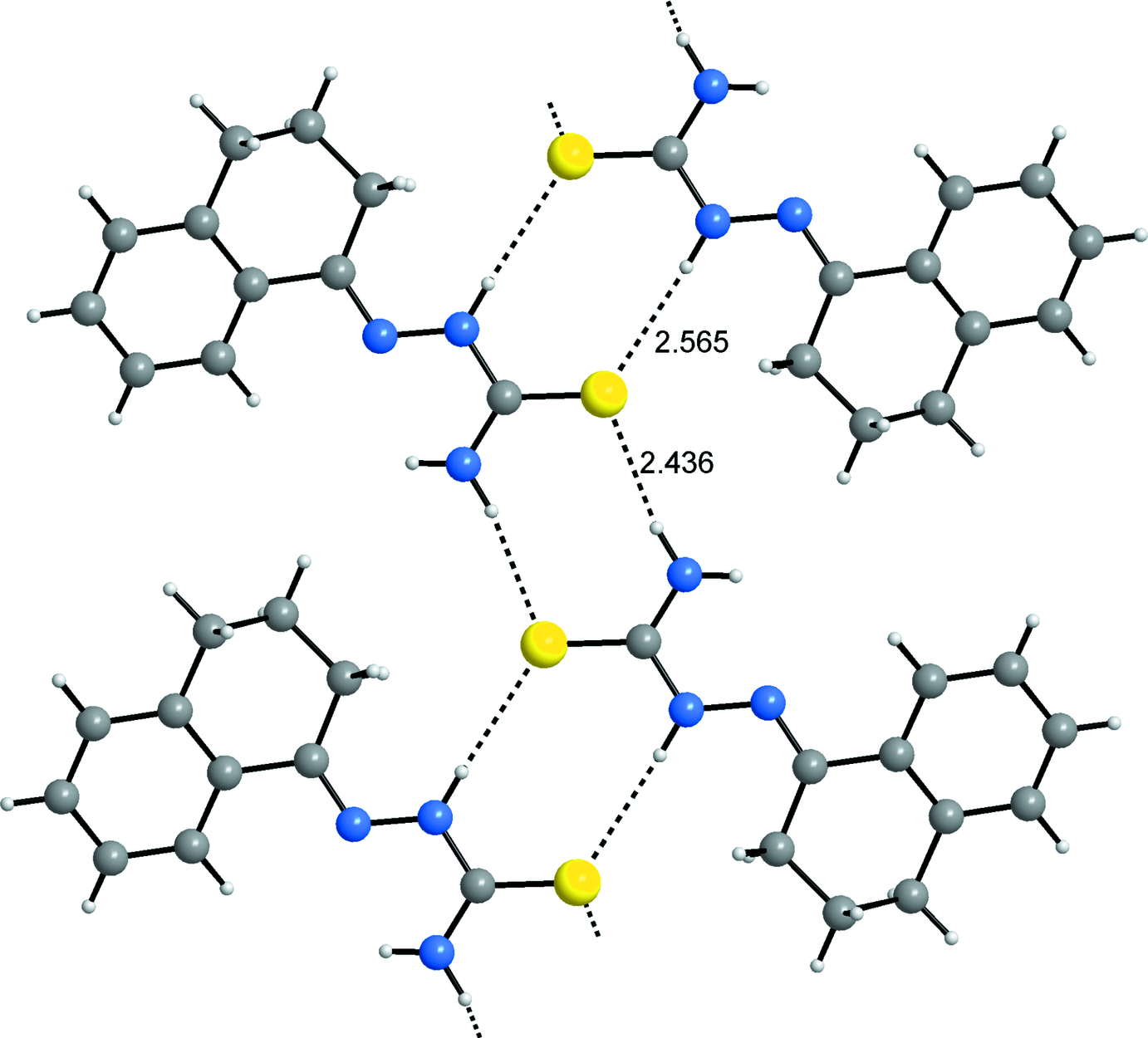

Supplement: Supplementary file 5 [file e-70-o1090-fig2.tif]

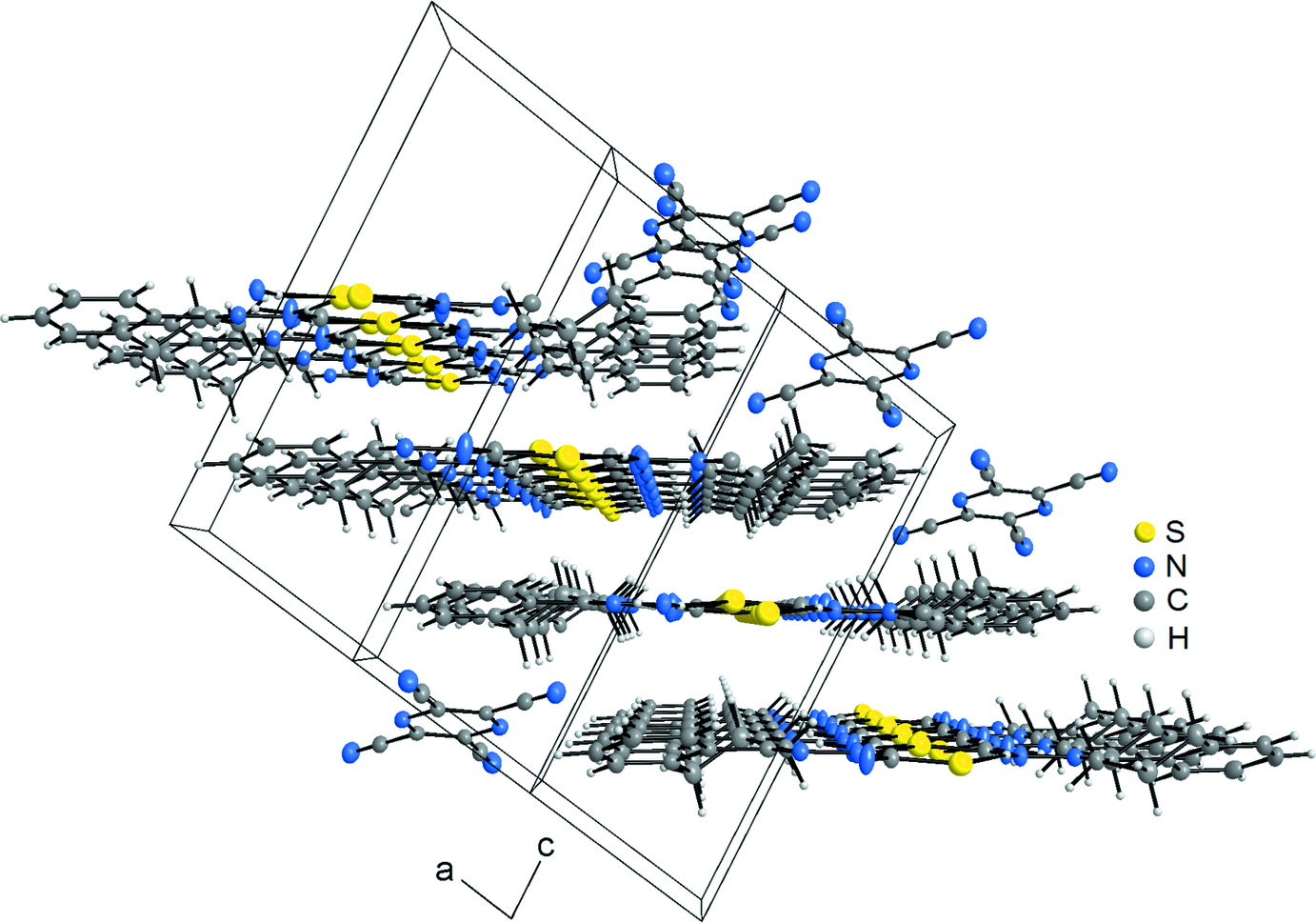

Supplement: Supplementary file 6 [file e-70-o1090-fig3.tif]

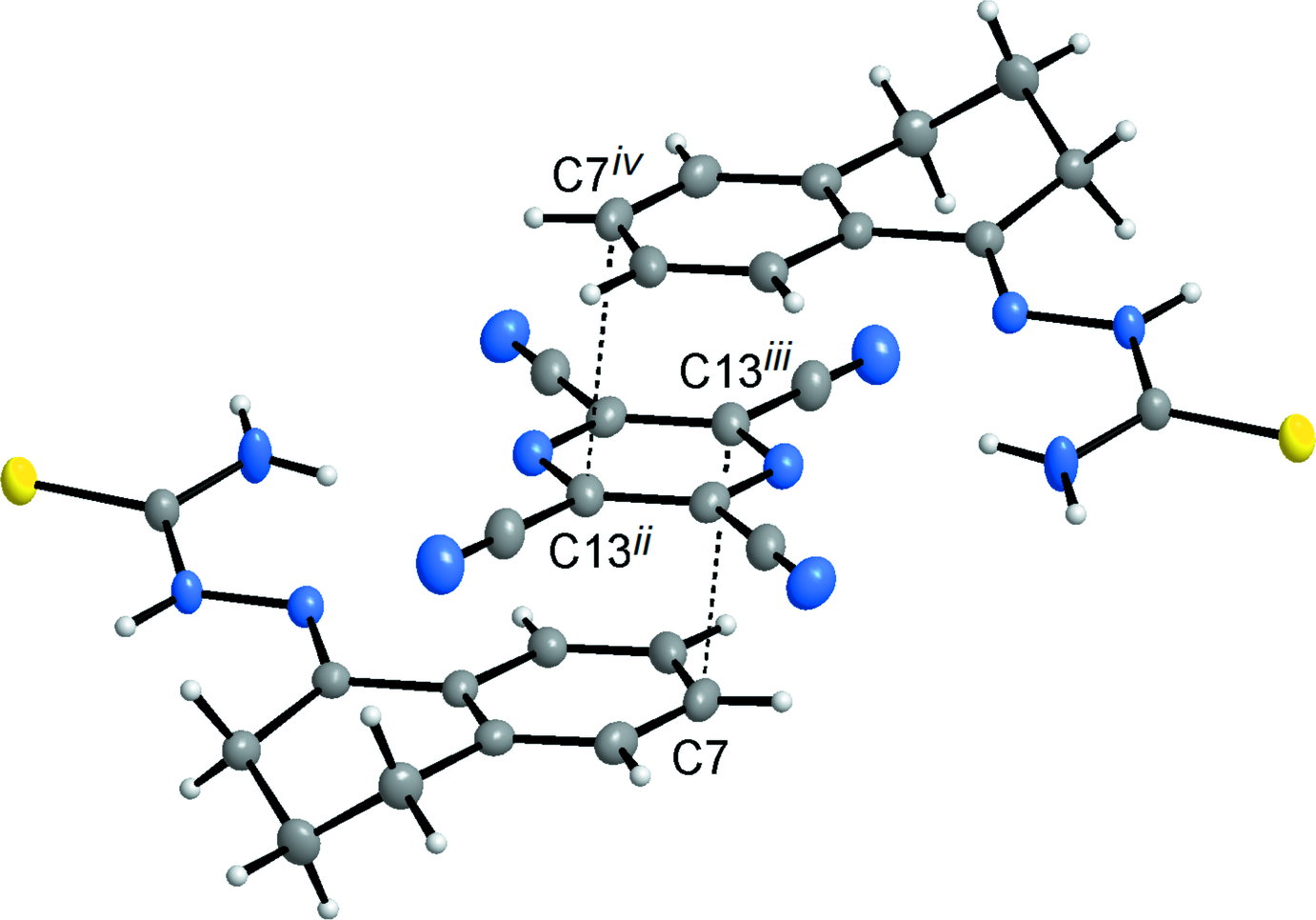

Supplement: Supplementary file 7 [file e-70-o1090-fig4.tif]

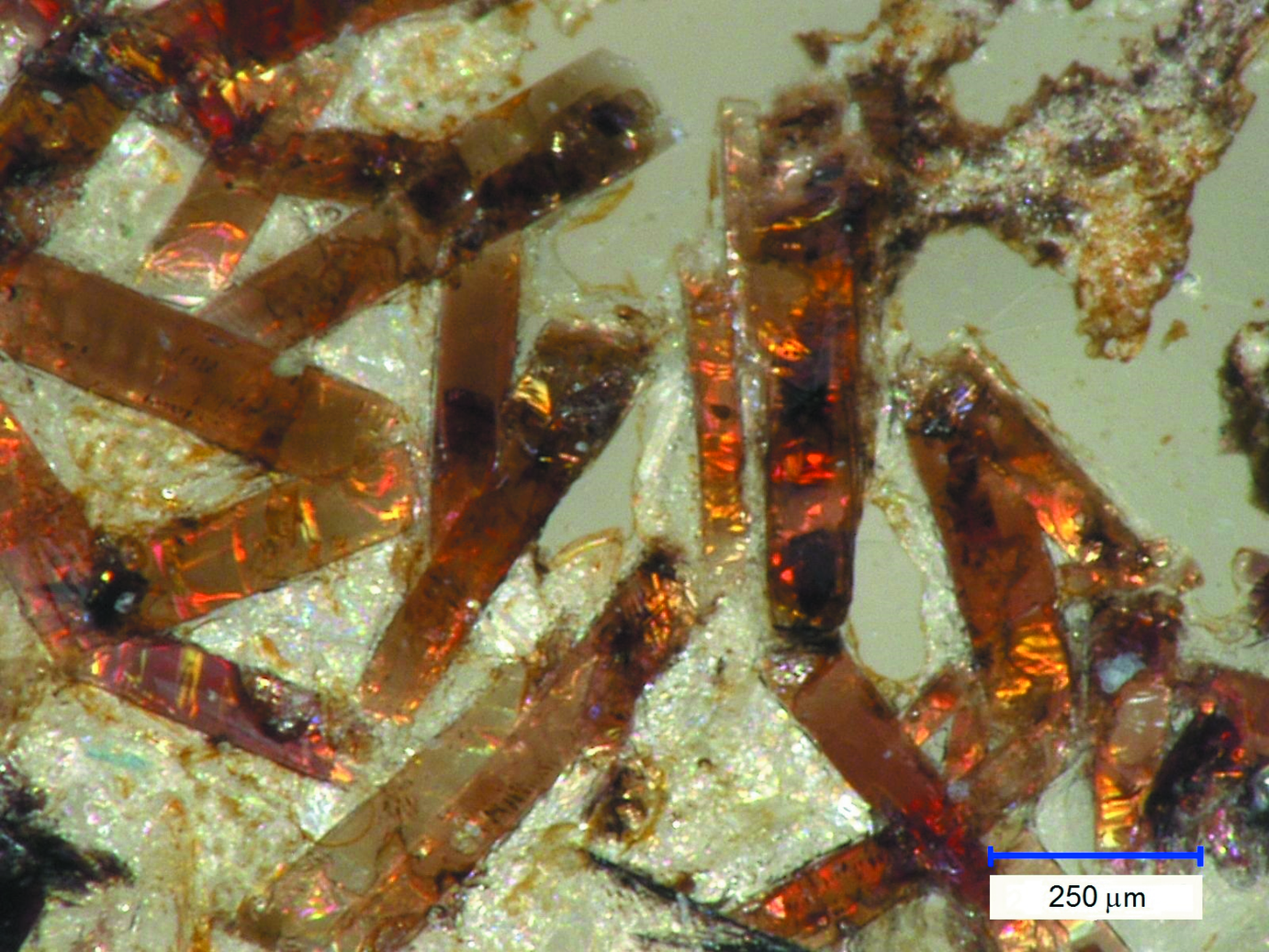

Supplement: Supplementary file 8 [file e-70-o1090-fig5.tif]

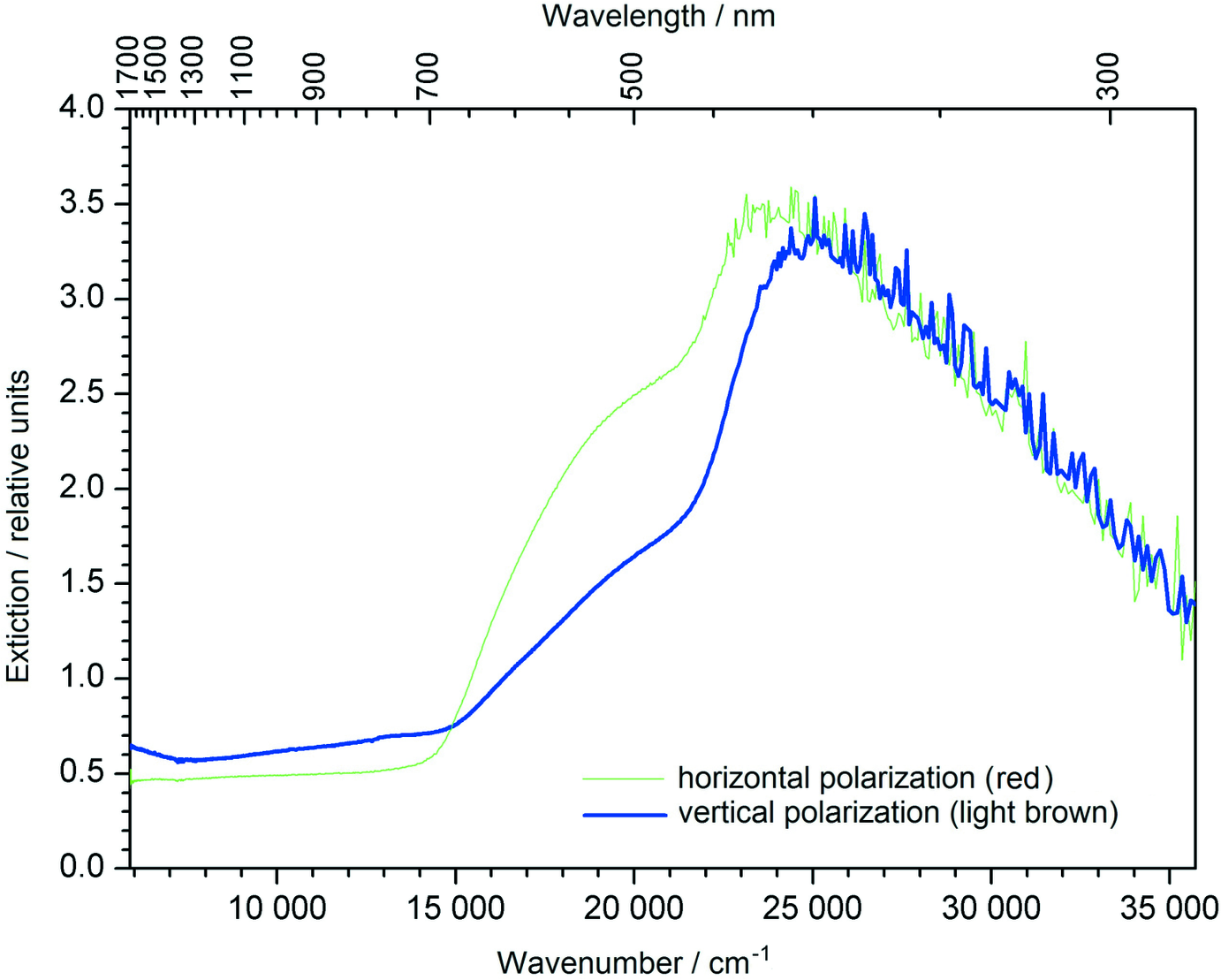

Supplement: Supplementary file 9 [file e-70-o1090-fig6.tif]
